# Fish Consumption and the Risk of Depression: A Systematic Review and Meta-Analysis of Observational Studies

**DOI:** 10.3390/nu17243965

**Published:** 2025-12-18

**Authors:** Eunje Kim, Youjin Je

**Affiliations:** Department of Food and Nutrition, Kyung Hee University, Seoul 02447, Republic of Korea; bile176@khu.ac.kr

**Keywords:** depression, fish, public health, nutrition, epidemiology, meta-analysis, systematic review

## Abstract

**Background/Objectives**: This systematic review and meta-analysis of observational studies aimed to assess the association between fish consumption and the risk of general and pregnancy-related depression, with implications for public health promotion. **Methods**: We retrieved 5074 articles from PubMed and Embase through November 2023 and included 35 observational studies in the analysis. We synthesized effect estimates as relative risks (RRs) with corresponding 95% confidence intervals (CIs) using a random-effects model. Additional dose–response analyses and stratified subgroup analyses were performed. **Results**: A significant inverse association was found between fish consumption and depression risk (RR = 0.79, 95% CI: 0.73 to 0.86). A similar association was observed for pregnancy-related depression (RR = 0.78, 95% CI: 0.69–0.89). Stratified analyses showed that only studies with fish intake ≥ 68.4 g/day demonstrated a statistically significant reduction in depression risk (RR = 0.75, 95% CI: 0.67–0.84), whereas studies with lower intake (<68.4 g/day) showed no significant association (RR = 0.83, 95% CI: 0.69–1.01), suggesting a potential threshold effect. Dose–response analysis further supported a 6% reduction in depression risk per 15 g/day increase in fish intake. **Conclusions**: This meta-analysis supports fish consumption as a modifiable factor for depression prevention, with ≥68.4 g/day as a possible threshold, potentially informing dietary guidelines and public health strategies.

## 1. Introduction

According to the World Health Organization (WHO), depression represents a major global public health burden, affecting approximately 280 million people worldwide and contributing substantially to suicide-related mortality [1]. Common mental disorders such as depression and anxiety are estimated to cost the global economy $1 trillion annually, mainly from lost productivity [2]. The burden of depression has further increased during and after the corona virus disease 2019 (COVID-19) pandemic [3,4,5]. However, due to poor compliance, side effects, and high recurrence of antidepressants, current treatment for depression remains unsatisfactory [6]. Given the public health impact of depression, identifying modifiable risk factors is crucial. Several studies have shown that depression is associated with various lifestyle-related factors, including levels of physical activity [7,8], alcohol consumption [9], and dietary patterns such as meat consumption [10] and intake of fruits and vegetables [11].

Among dietary factors, evidence from previous meta-analyses suggests that higher fish consumption is linked to a lower risk of depression [12,13,14]. Despite these findings, quantitative evidence specifically addressing pregnancy-related depression in relation to fish intake remains limited. Depression is known to be more prevalent in women (5.1%) than in men (3.6%) [15], and even during the pandemic, women were found to have higher levels of depression than men [3]. Approximately 20% of women experience clinical depression during their lifetime [16], and postpartum depression affects one in five women after giving birth [17].

Fish provides substantial amounts of omega-3 fatty acids, and prior quantitative reviews have indicated a potential protective relationship between omega-3 intake and depression risk [12,13,14,18]. Pregnancy is accompanied by increased nutritional demands to support fetal development and maternal metabolic adaptation [19]. In particular, the need for long-chain omega-3 polyunsaturated fatty acids (PUFAs) rises during pregnancy and lactation compared with non-pregnant periods [20]. Therefore, depletion of omega-3 PUFAs during pregnancy may increase the risk of depression.

Several cross-sectional [21,22,23,24,25,26,27,28,29,30,31,32,33,34,35,36,37] and cohort studies [38,39,40,41,42,43,44,45,46,47,48,49,50,51,52,53,54], as well as a case–control study [55], have investigated the link between fish intake and depression risk, but the results have been inconsistent. Notably, 5 studies specifically investigating fish consumption in relation to pregnancy-related depression also revealed inconsistent findings. This is of particular concern, as pharmacologic treatment may not be a preferred or feasible option for many pregnant or breastfeeding women. Given the heterogeneity of previous findings, we performed an updated meta-analysis integrating recent observational evidence, including pregnancy-specific populations. In addition, dose–response relationship and potential intake thresholds associated with reduced depression risk were explored. These findings aim to provide updated evidence to support dietary recommendations and public health strategies for depression prevention.

Compared with previous meta-analyses, this study incorporates the most recent observational evidence, includes pregnancy-related depression as a distinct subgroup, examines differential intake thresholds based on the magnitude of intake contrast between comparison groups, and performs expanded stratified analyses across population subgroups. These additions enhance the current understanding of fish consumption in relation to depression.

## 2. Materials and Methods

### 2.1. Search Strategy

Relevant studies published through November 2023 were identified by searching the PubMed and Embase databases. The following terms were used in the search: “(fish OR fish meat OR seafood)” AND “(depression OR depressive disorder OR depressed mood OR depressive symptom)”. Reference lists of relevant reviews and included articles were also manually screened to capture further eligible studies.

### 2.2. Study Criteria

Studies were included in the meta-analysis based on predefined eligibility criteria: (1) studies in observational study design, including cross-sectional, case–control, and cohort studies; (2) fish intake was considered the main exposure of interest; (3) depressive outcomes, including pregnancy-related depression, were assessed; (4) effect estimates were reported as relative risks with corresponding confidence intervals, or sufficient information was available for their calculation. When multiple publications were based on the same cohort or population, a single study was retained, prioritizing the most recent publication or, where applicable, the one with the largest sample size. Overlapping populations were identified by comparing cohort names, study registries, recruitment periods, and participant characteristics.

### 2.3. Extraction of Data

Two investigators (E.K. and Y.J.) independently extracted data from the included studies, following the Preferred Reporting Items for Systematic Reviews and Meta-Analyses (PRISMA) recommendations [56]. The following information was systematically collected from each eligible study: bibliographic information; study design; follow-up characteristics; study setting; participant demographics; baseline age; numbers of cases and participants; methods used to assess dietary intake and outcomes; fish intake categories with corresponding relative risks (RRs) and 95% confidence intervals (CIs); covariates included in the adjusted models. When multiple models were presented, we extracted the most fully adjusted model, defined as the model incorporating the most comprehensive set of confounders. Regarding the harmonization of depression measurements, although the included studies used different validated instruments to assess, all tools captured depressive symptoms or depressive disorder based on standardized and widely accepted methodologies. Therefore, for consistency across studies, we extracted the primary depression outcome reported in each study.

### 2.4. Quality Assessment

The methodological quality of the studies included in this meta-analysis was evaluated using the Newcastle–Ottawa Scale. Cross-sectional studies were evaluated across three domains—selection, comparability, and outcome—with a maximum possible score of 10 points. Cross-sectional studies with a total score of 8 points or higher were classified as high methodological quality. Case–control and cohort studies were assessed using a three-domain scale encompassing selection, comparability, and outcome, with a maximum score of 9 points. Studies with a total score of 7 points or above were considered to have high methodological quality.

### 2.5. Statistical Analysis

All statistical analyses were performed using STATA/SE software (version 14.0; StataCorp LP, College Station, TX, USA). RRs and beta coefficients and their associated 95% CIs in each study were used to examine the association between fish consumption and depression risk. When studies reported hazard ratios (HRs) or odds ratios (ORs) rather than RRs, these measures were treated as equivalent estimates, a common approach in meta-analyses of observational studies when event rates are low [57]. For comparisons between the highest and lowest categories of fish intake, the natural logarithm values of the RRs from the original studies were pooled using the DerSimonian and Laird random-effects models [58]. Pooled results were displayed using forest plots where the effect estimates of individual studies were presented as the size of data markers (squares), and the pooled RR was presented as the diamond. The Q statistics [59] and the *I*^2^ statistics [60] were used to assess the statistical heterogeneity between studies. The heterogeneity assumption was considered significant when the *p* value was below 0.05.

A dose–response meta-analysis examining intake of fish in relation to depression risk was additionally performed using a two-stage generalized least-squares trend estimation approach [61,62]. Data required for dose–response modeling included fish intake levels, numbers of cases and participants, and RRs with corresponding 95% CIs across at least three exposure categories. The median or mean intake value reported for each category was used to represent the corresponding exposure dose. When intake categories were defined by ranges, the midpoint between the lower and upper boundaries was calculated and applied as the exposure value. For open-ended intake categories, the lower boundary of the lowest category was set to zero, whereas the width of the highest open-ended category was assumed to match that of the adjacent category. To ensure comparability across studies using different dietary assessment methods, fish intake was standardized to a common unit. When intake was expressed as servings per week or month, values were converted to grams per day using a standard portion size of 105 g per serving [63]. In addition, a nonlinear relationship was explored using restricted cubic spline model with four knots placed at the 5th, 35th, 65th, and 95th percentiles.

To explore sources of variability in effect estimates, we conducted subgroup analyses stratified by geographic region (Europe, Asia, America, Multicenter, Oceania), whether it is related to pregnancy (subjects who were pregnant or have recently given birth vs. general population), study design (cross-sectional, case–control, cohort), gender (both men and women, men, women), age (only elderly population vs. general population), intake level of the highest exposure category (higher or lower than the median), and the difference between intake between the highest lowest group (higher or lower than the median). Additionally, publication bias was evaluated using Begg and Mazumdar’s test [64] and Egger’s regression test [65]. Statistical significance was defined as a two-tailed *p* value below 0.05.

## 3. Results

### 3.1. Literature Search

**Figure 1:**
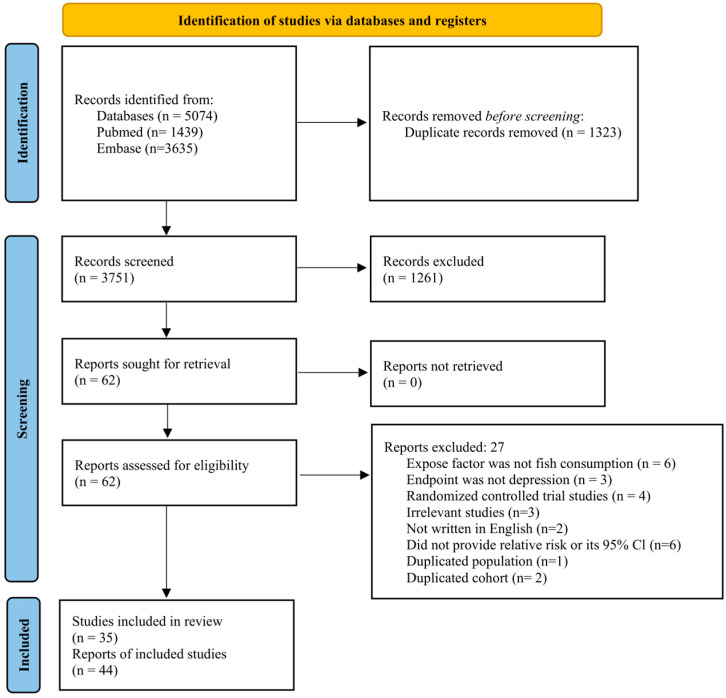
PRISMA flow diagram. Method for the selection of studies for the analysis.

The process of literature identification and study selection is summarized in Figure 1. The database search identified a total of 5074 records, including 1439 from PubMed and 3635 from Embase. After excluding 1323 duplicate articles, 3751 articles remained. The titles and abstracts of the retrieved 3751 articles were screened, resulting in removal of 3689 articles (reviews, letters, animal studies, laboratory studies, and those irrelevant to the current study). The remaining 62 articles were screened by full-text review. During full-text screening, additional studies were excluded for reasons including non-relevant exposure or outcome definitions, inappropriate study design, non-English language, insufficient effect estimates, or due to overlapping population data. As a result, 35 observational study articles, including 44 studies, met our criteria and were included in the meta-analysis.

### 3.2. Study Characteristics

Key characteristics of the included studies—comprising 20 cross-sectional studies, 1 case–control study, and 23 cohort studies—are summarized in Table 1. Regarding geographic distribution, 18 studies were conducted in Europe, 7 in the Americas, 13 in Asia, and 3 were conducted in Oceania, while 2 were multicenter studies. Dietary intake was predominantly assessed using Food Frequency Questionnaires (FFQ), except for one study that used 24 h dietary recall and one study that used standardized questions. Various methods were used for diagnosing depression, including 21-item Beck Depression Inventory (BDI), hospital treatment, Hopkins Symptom Check List-25 subscale (HSCL-25), diagnosis by a professional, Welsh Pure Depression subscale of the Minnesota Multiphasic Personality Inventory, antidepressant or lithium prescription, self-reported physician diagnosis of depression, anxiety or stress or use of antidepressant medication or tranquilizers, post-partum depression hospital admission or medicament prescription, the Center for Epidemiological Studies Depression Scale (CES-D), the Edinburgh Postnatal Depression Scale (EPDS), Geriatric Depression Scale (GDS), the Munich version of the Composite International Diagnostic Interview (M-CIDI), the Composite International Diagnostic Interview Short-Form (CIDI-SF), the Center for Epidemiological Studies Depression Scale, Korean version (CES-D-K), Diagnostic and Statistical Manual of Mental Disorders, Fourth Edition (DSM-IV), depression, anxiety and stress scale questionnaire (DASS), and International Classification of Diseases 10th edition (ICD-10). In most studies, age, gender, education level, and body mass index (BMI) were adjusted. Based on the Newcastle-Ottawa quality assessment scale, quality scores ranged from 6 to 10 for cross-sectional studies, 8 for the case–control study, and 6 to 9 for cohort studies. Overall, all but four studies met the criteria for high quality. Detailed quality assessment results for individual studies are provided in Supplementary Tables S1–S3.

### 3.3. Pooled Relative Risk Estimates for Comparisons Between High and Low Intakes

**Figure 2:**
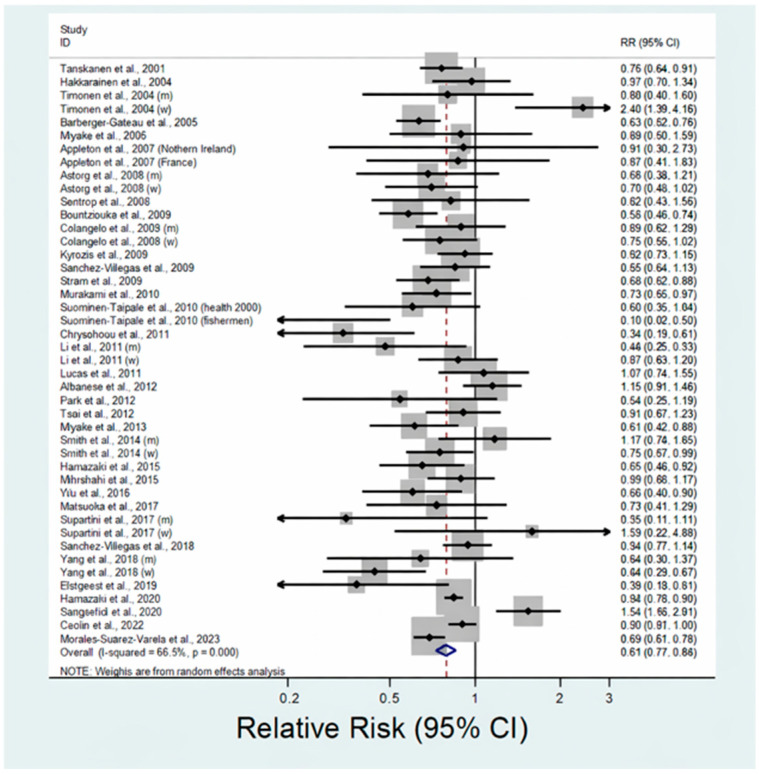
Forest plot of relative risks (RRs) and 95% confidence intervals (CIs) for depression comparing the highest versus lowest categories of fish consumption. Squares indicate study-specific effect estimates (with size proportional to study weight), horizontal lines represent 95% CIs, and the diamond denotes the pooled estimate derived from a random-effects model. The solid vertical line indicates RR = 1.0. Results reported separately for men (m) and women (w) are indicated accordingly. References: [21,22,23,24,25,26,27,28,29,30,31,32,33,34,35,36,37,38,39,40,41,42,43,44,45,46,47,48,49,50,51,52,53,54,55].

Figure 2 presents the results of the meta-analysis based on the most fully adjusted relative risks comparing the highest and lowest categories of fish intake across studies. Across 44 studies, higher fish consumption was associated with a lower risk of depression, with a pooled relative risk of 0.79 (95% CI: 0.73–0.86) and moderate between-study heterogeneity (*I*^2^ = 66.5%).

Several RRs obtained from the subgroup meta-analysis were shown in Table 2. Stratifying by region, the pooled RRs for each region were 0.75 (95% CI: 0.64–0.87) in Europe, 0.75 (95% CI: 0.63–0.90) in Asia, 0.88 (95% CI: 0.80–0.96) in America, 0.88 (0.53–1.45) in multicenter studies, and 0.87 (95% CI: 0.71–1.08) in Oceania. When stratified by whether the depression was related to pregnancy or not, the pooled RR for pregnancy-related depression was 0.78 (95% CI: 0.69–0.89), and the pooled RR of studies on general depression was 0.79 (95% CI: 0.72–0.87). Stratifying by study design, the pooled RRs for each study design were 0.72 (95% CI: 0.63–0.83) in cross-sectional studies, 0.54 (95% CI: 0.25–1.19) in case–control study, and 0.85 (95% CI: 0.78–0.93) in cohort studies. Stratifying by gender, the pooled RR of men and women both was 0.77 (95% CI: 0.68–0.87), while the pooled RR was 0.81 (95% CI: 0.68–0.99) in men, and 0.81 (95% CI: 0.71–0.93) in women. When stratified by whether the study was for only elderly population or was for general population, the pooled RRs were 0.75 (95% CI: 0.62–0.91) and 0.80 (95% CI: 0.73–0.88), respectively. Additional subgroup analyses were performed according to the median intake level of the highest fish consumption category (68.4 g/day). For studies with median intake levels below 68.4 g/day, the pooled relative risk was 0.83 (95% CI: 0.69–1.01), whereas studies with intake levels at or above 68.4 g/day showed a pooled relative risk of 0.75 (95% CI: 0.67–0.84). When stratified by the median difference in fish intake between the highest and lowest categories (49.35 g/d), pooled relative risks were 0.85 (95% CI: 0.72–1.02) for smaller contrasts and 0.72 (95% CI: 0.63–0.83) for larger contrasts.

### 3.4. Dose–Response Analyses

Both linear and nonlinear dose–response meta-analyses were performed using data from 7 cross-sectional studies and 7 cohort studies. Linear dose–response analysis indicated that each 15 g/d increase in fish intake was associated with a pooled RR of 0.94 (95% CI: 0.92–0.96). No evidence of a nonlinear association between fish intake and depression risk was observed (*p* = 0.204). When stratified by geographic region, pooled relative risks were 0.92 (95% CI: 0.88–0.96) in Europe, 0.94 (95% CI: 0.92–0.97) in Asia, and 0.89 (95% CI: 0.75–1.04) in America. Stratification by pregnancy status yielded pooled relative risks of 0.93 (95% CI: 0.89–0.98) for pregnancy-related depression and 0.94 (95% CI: 0.91–0.96) for depression in the general population. By study design, pooled RRs were 0.93 (95% CI: 0.90–0.96) for cross-sectional studies and 0.94 (95% CI: 0.92–0.97) for cohort studies. When stratified by sex, pooled RRs were 0.95 (95% CI: 0.93–0.97) for mixed-sex populations, 0.90 (95% CI: 0.80–1.02) for men, and 0.91 (95% CI: 0.87–0.96) for women. Stratification by age group showed pooled RRs of 0.96 (95% CI: 0.93–0.99) in studies of older adults and 0.93 (95% CI: 0.90–0.95) in studies of the general population.

### 3.5. Meta-Analysis and Sensitivity Analysis

Analysis of 44 studies revealed moderate between-study heterogeneity (*I*^2^ = 66.5%, *p* = 0.0). Univariate meta-regression analyses were performed for region, pregnancy status, sex, and study design, but none of these variables significantly explained the observed heterogeneity (*p* > 0.05).

Leave-one-out sensitivity analyses identified 7 studies out of 44 studies [21,22,23,25,31,37,53] as contributors to the observed between-study heterogeneity. Notably, these studies commonly categorized fish consumption using simplified, dichotomous definitions (e.g., intake vs. non-intake or infrequent vs. frequent) rather than multiple or quantitative intake categories. After further excluding these studies, the heterogeneity (*I*^2^ = 29.6%, *p* = 0.048) was decreased and the result 0.80 (95% CI: 0.75–0.85) remained significant.

### 3.6. Publication Bias

For comparisons between the highest and lowest fish intake categories, publication bias was not indicated by either Begg’s test (*p* = 0.140) or Egger’s test (*p* = 0.239). Egger’s regression test did not suggest the presence of publication bias in the dose–response meta-analysis (*p* = 0.207). In contrast, Begg and Mazumdar’s rank correlation test indicated potential publication bias (*p* = 0.006). These results suggest that the dose–response findings warrant cautious interpretation.

## 4. Discussion

This meta-analysis of observational evidence indicates that higher fish consumption is associated with a lower risk of depression. Compared with individuals in the lowest intake category, those with the highest fish consumption exhibited an approximately 21% lower risk of depression. Although the overall heterogeneity was moderate to high (*I*^2^ = 66.5%), the direction of associations remained consistent across subgroup and sensitivity analyses, supporting the robustness of the observed relationship. The observed heterogeneity may partly reflect methodological differences across studies, including variation in depression assessment instruments and differences in how fish consumption was categorized. Notably, sensitivity analyses indicated that studies contributing most to between-study heterogeneity commonly employed simplified, dichotomous definitions of fish intake (e.g., intake vs. non-intake) rather than multiple or quantitative intake categories. Importantly, exclusion of these studies substantially reduced heterogeneity while the overall inverse association remained statistically significant, indicating that the main conclusions are robust. Findings from the dose–response analysis further reinforced the inverse relationship between fish intake and depression risk. Although the included studies encompassed heterogeneous populations (e.g., elderly adults, general adults, and pregnant women), pooling these populations enhances the generalizability of the findings. Moreover, subgroup analyses stratified by key demographic characteristics yielded generally consistent associations, supporting the validity of the combined analyses.

When analyses were stratified by geographic region, a pronounced inverse association between fish consumption and depression risk was observed in studies conducted in Europe and Asia. Specifically, pooled relative risks were 0.75 (95% CI: 0.64–0.87) for Europe and 0.75 (95% CI: 0.63–0.90) for Asia. This pattern may partly reflect higher habitual fish consumption in European and Asian populations compared with populations in the Americas and Oceania [66]. The stronger protective association observed in Europe and Asia, regions with higher habitual fish intake, further supports the hypothesis that greater fish consumption may help reduce depression risk. The observed regional differences may also reflect broader dietary contexts rather than the effect of fish intake alone. Previous cross-national evidence suggests that the mental health effects of dietary factors may depend on whether intake is sufficient within the overall dietary context and nutritional adequacy [67,68]. In this context, populations in Europe and Asia, where fish is more commonly integrated into habitual dietary patterns, may be more likely to achieve intake levels at which protective associations become detectable. Conversely, in regions with lower baseline intake or different dietary structures, similar amounts of fish consumption may represent a relatively small contribution to overall diet and thus be insufficient to confer measurable benefits. Consistent with this interpretation, subgroup analysis based on the median intake level of the highest fish consumption category indicated no significant risk reduction effect in studies with fish intake less than 68.4 g/d, whereas a significant 25% risk reduction was found in studies with fish intake more than 68.4 g/d. These results suggest that relatively low levels of fish consumption may not be sufficient to confer a protective association against depression. Additional subgroup analyses based on the median difference in fish intake between the highest and lowest categories further supported the primary findings. In studies with intake contrasts exceeding 49.35 g/d between the highest and lowest categories, fish consumption was associated with a 28% lower risk of depression, whereas no significant association was observed in studies with smaller intake contrasts. Compared with previous meta-analyses, which primarily contrasted with the highest versus lowest categories of fish intake without considering absolute intake levels, the present study may offer complementary information by examining approximate intake levels and intake contrasts derived from observational data. Our findings indicate that protective associations tended to be more consistently observed at relatively higher levels of fish consumption, which may partly account for the heterogeneity and inconsistent results reported in earlier studies.

Several mechanisms may underlie the association between fish consumption and a reduced risk of depression. Rather than a single causal pathway, fish consumption may influence depression risk through multiple, interrelated biological processes involving neuroinflammation, neurotransmitter regulation, and cardiovascular health. Fish provides a major dietary source of omega-3 PUFAs, and previous studies have reported inverse associations between omega-3 fatty acid intake and depression risk [12,13,14,18], suggesting that these omega-3 PUFAs derived from fish consumption may contribute to the observed reduction in depression risk. Omega-3 PUFAs have been reported to influence serotonergic nervous system function [69], and serotonin, which can be upregulated by omega-3 PUFAs, has been shown to be inversely associated with depression [53]. Omega-3 PUFAs may also influence depression risk through modulation of dopaminergic pathways and regulation of corticotropin-releasing factor activity [70]. Recent studies have shown that oxidative stress [71], immune system functioning, and inflammation [72] may contribute to the pathophysiology of depression, and several studies have reported that omega-3 PUFAs have the potential to regulate oxidative stress [73,74], immune system function, and inflammation [73,75,76]. Depression has also been closely associated with cardiovascular conditions, such as coronary heart disease and stroke, with evidence suggesting shared and bidirectional pathophysiological pathways [77,78,79]. Chronic systemic inflammation, oxidative stress, and immune system function have been proposed as common mechanisms underlying both depressive disorders and cardiovascular dysfunction [80]. Fish-derived nutrients, particularly omega-3 PUFAs, may exert beneficial effects on both neuropsychiatric and cardiovascular health through overlapping biological pathways [75,76], which may partly explain the observed association between fish consumption and depression risk.

In addition to omega-3 PUFAs, various nutrients found in fish may also play a role in reducing depression risk. Several studies have reported that vitamin D has a negative association with depression risk [81,82], as vitamin D works in cooperation with omega-3 PUFAs to regulate circadian rhythms and synthesis of neurotransmitters [74]. Additionally, selenium contained in fish can act as an antioxidant and prevent methylmercury toxicity [83], which may contribute to the prevention of depression [84]. Moreover, it has been reported that the nutrients found in fish, including vitamin B2, vitamin B6, vitamin B12 [85], vitamin E [86], and folate [87] can contribute to reducing the risk of depression.

Our study also demonstrated a significant reduction in pregnancy-related depression risk with higher fish consumption. This finding aligns with several randomized controlled trials reporting inverse associations between omega-3 fatty acid supplementation and pregnancy-related depression [88,89,90]. The underlying mechanisms may involve changes in maternal brain lipid composition and neuroplasticity during pregnancy. The brain, particularly the gray matter, is rich in omega-3 PUFAs, with arachidonic acid accounting for 8–10% and docosahexaenoic acid (DHA) accounting for 9–14% [91,92]. One study reported a decrease in gray matter volume in pregnant women [93]. In the case of mothers with a DHA-deficient diet, sufficient DHA supply for the fetus was found to be supplied from the mother’s brain, which lowered maternal DHA levels [94]. Through those findings, it can be assumed that the brain volume reduction during pregnancy could be partly due to the loss of PUFAs. DHA is highly concentrated in synaptic neuronal membranes and is a critical source for synaptic function, which plays a role in mood disorders [95]. Considering these mechanisms and findings from our study, it can be inferred that fish consumption may be crucial for pregnant women to prevent depression.

Beyond biological plausibility, our findings have important public health implications. Identifying a potential intake level at which protective associations were more consistently observed (≥68.4 g/day or an intergroup difference of ≥49.35 g/day) provides a quantifiable reference point that may inform future dietary research and public health discussions. However, these values were derived from median intake levels across included studies rather than from biological or clinical evidence, and therefore should be interpreted cautiously as hypothesis-generating rather than prescriptive targets. While further research is required to validate these thresholds, they may inform evidence-based dietary recommendations for depression prevention, particularly in populations with low habitual fish consumption. Such evidence-based recommendations could be incorporated into national nutrition policies, workplace wellness programs, and prenatal care guidelines. As depression imposes a substantial global economic burden and treatment efficacy remains limited, dietary strategies based on achievable intake targets represent a practical and scalable component of comprehensive prevention frameworks. In addition, these findings highlight the need for future intervention studies and dietary guidelines to consider absolute intake levels, rather than relying solely on relative intake categories, when evaluating the mental health effects of fish consumption.

This study has several strengths. To our knowledge, this meta-analysis represents the most comprehensive and up-to-date synthesis of observational evidence examining the association between fish consumption and depression risk. The inclusion of 44 datasets from diverse populations across multiple regions enhances the generalizability and reliability of our findings. We also conducted extensive subgroup analyses, including the first to address pregnancy-related depression, and dose–response analyses to further characterize the association. Moreover, subgroup analyses identified empirically derived intake levels (68.4 g/day or an intergroup difference of 49.35 g/day) above which protective effects were more consistently observed.

Despite these strengths, several limitations of this study should be noted. First, although most of the studies included in the meta-analysis were adjusted for a variety of potential confounders, residual confounding related to unmeasured or inadequately measured factors cannot be fully excluded, given that this meta-analysis relied on observational study designs. In addition, dietary intake in most included studies was assessed using self-reported instruments such as FFQs, which are subject to measurement error and may lead to attenuation of the observed associations. Second, methods used to assess dietary intake and diagnose depression varied across the included studies. Third, limited information on fish types and the amounts of specific fish consumed was available in many included studies, which restricted the ability to identify associations between particular fish species and depression risk. Fourth, because several included studies were cross-sectional, reverse causation cannot be ruled out; individuals with depressive symptoms may reduce their appetite or consumption of fish, potentially biasing associations. Fifth, cultural differences in cooking practices, such as frying versus raw or steamed fish consumption, as well as potential exposure to contaminants, were not consistently accounted for in the included studies and may have influenced the observed associations. Finally, as the meta-analysis was restricted to articles published in English, the potential for language bias cannot be excluded.

## 5. Conclusions

In conclusion, the results of this meta-analysis indicate that higher fish consumption is associated with a 21% reduction in the risk of depression. Protective associations were consistent across general and pregnancy-related populations, with effects becoming more pronounced at intake levels of approximately 68.4 g/day or when the intergroup difference exceeded 49.35 g/day. These findings support the potential role of fish as a potential dietary approach for depression prevention and provide a quantifiable target for dietary guidelines and public health initiatives. Future studies should explore the effects of specific fish species, preparation methods, and long-term dietary patterns to refine evidence-based recommendations for mental health promotion.

## Figures and Tables

**Table 1:** Characteristics of observational studies analyzed in the meta-analysis in relation to fish intake and depression risk.

Author, Year; Study Design (Follow-Up)	Country	Participants Characteristics	No. of Subjects	Exposure Assessment	Outcome Measure	Amount of Fish Intake	RR or β Coefficient (95% CI)
Tanskanen et al., 2001 [35]; cross-sectional	Finland	General population; age: 25–64	3204	FFQ	BDI	Rare eaters vs. regular eaters	1.31 (1.10, 1.56)
Hakkarainen et al., 2004 [42]; cohort (5–8 y)	Finland	General population; age: 50–69	29,133	FFQ	Hospital treatment	Quartile3 vs. Quartile1	0.97 (0.70, 1.33)
Timonen et al., 2004 [53]; cohort (31 y)	Finland	General population; age: <31	5689	FFQ	HSCL-25 and diagnosis by medical doctor	regular eaters vs. rare eaters	Men: 0.8 (0.4, 1.6) Women: 2.4 (1.4, 4.2)
Barberger-Gateau et al., 2005 [22]; cross-sectional	France	Community dwellers; age: ≥65	9280	FFQ	CES-D	>1 time/week vs. 1 time/week	0.63 (0.52, 0.75)
Miyake et al., 2006 [49]; cohort (2–9 m)	Japan	Pregnant women; age: <32	865	FFQ	EPDS	72.9 g/day vs. 23.1 g/day	0.89 (0.50, 1.59)
Appleton et al., 2007 [38]; cohort (5 y)	Northern Ireland and France	General population (men); age: 50–59	10,602	FFQ	Welsh Pure Depression subscale	Linear term	Northern Ireland: −0.09 (−2.25, −0.01) France: −0.14 (−2.73, −1.17)
Astorg et al., 2008 [39]; cohort (2 y)	France (SU.VI.MAX cohort study)	General population; age 35–60	3748	24 h dietary recall	Antidepressant	Men: 87.9 ± 29.5 g/day vs. 14.9 ± 8.9 g/day Women: 71.6 ± 24.8 g/day vs. 10.7 ± 7.1 g/day	Men: 0.68 (0.38, 1.21) Women: 0.70 (0.48, 1.02)
Sontrop et al., 2008 [32]; cross-sectional	Canada	Pregnant women (10- and 22-week gestation)	2061	FFQ	CES-D	≥1 serving/week vs. <1 serving/week	−0.2 (−0.9, 0.4)
Bountziouka et al., 2009 [23]; cross-sectional	Greece and Cyprus	Elderly general population; age ≥65	1190	FFQ	GDS (self-report)	Linear term (1 portion of fish increase per week)	0.58 (0.45, 0.73)
Colangelo et al., 2009 [40]; cohort (10 y)	US	General population; age 24–42	3317	FFQ	CES-D	Quartile5 vs. Quartile1	Men: 0.89 (0.62, 1.28) Women: 0.75 (0.55, 1.01)
Kyrozis et al., 2009 [44]; cohort (6–13 y)	Greece	Elderly general population; age ≥60	610	FFQ	GDS	Linear term	−0.08 (−0.30, 0.15)
Sánchez-Villegas, 2009 [50]; cohort (4.4 y)	Spain	General population; age 38 (mean)	10,094	FFQ	Self-reported physician diagnosis, antidepressant medication usage	Quartile5 vs. Quartile1	0.85 (0 64, 1.13)
Strøm et al., 2009 [52]; cohort (1 y)	Denmark	Women; age 25–40	54,202	FFQ	Hospital admission of post-partum depression, medicament prescription	0–3 g/day (1.1 g/day) vs. >30 g/day (38.0 g/day)	1.10 (0.87, 1.38)
Murakami et al., 2010 [29]; cross-sectional	Japan	Adolescents (school students); age 12–15	6517	FFQ	CES-D	29.1 g/1000 kcal vs. 9.1 g/1000 kcal	0.73 (0.55, 0.97)
Suominen-Taipale et al., 2010 [33]; cross-sectional	Finland	General population; age 45–74	5492	FFQ	M-CIDI	76 g/day vs. 11 g/day	0.6 (0.3, 0.9)
Suominen-Taipale et al., 2010 [33]; cross-sectional	Finland	Fishermen with their families	1265	FFQ	CIDI-SF	Quartile4 vs. Quartile1	0.1 (0.02, 0.5)
Chrysohoou et al., 2011 [25]; cross-sectional	Greece	Elderly population; age >65	673	FFQ	GDS (self-report)	≥3 times week vs. never/rare	0.34 (0.19, 0.61)
Li et al., 2011 [45]; cohort (10.6 y)	US	General population; age 25–74	5068	FFQ	CES-D	<1 time/week vs. ≥1 time/week	Men: 2.08 (1.08, 4.09) Women: 1.15 (0.83, 1.59)
Lucas et al., 2011 [46]; cohort (10 y)	US	Nurses (women); age 50–77	54,632	FFQ	Physician-diagnosed depression, antidepressant usage	≥5 times/week vs. 1 time/month	1.07 (0.74, 1.55)
Albanese et al., 2012 [21]; cross-sectional	Multicenter	Community dwellers; age ≥65	14,926	Standardized questions	ICD-10	Never vs. some days vs. most days	Never: 0.93 (0.78, 1.10) Some days: 1 (reference) Most days: 1.07 (0.85, 1.36)
Park et al., 2012 [55]; case–control	Korea	Patients diagnosed with a score ≥ 25 on the CES-D-K and controls without a chronic disease	80 patients and 88 controls	FFQ	CES-D-K	>9.62 serving/week vs. ≤2.57 serving/week	0.54 (0.19, 0.92)
Tsai et al., 2012 [54]; cohort (5 y)	Taiwan	Elderly population; age ≥65	1609	FFQ	CES-D	≥3 times/week vs. <3 times/week	0.91 (0.62, 1.14)
Miyake et al., 2013 [27]; cross-sectional	Japan	Pregnant women	1745	FFQ	CES-D	71.7 g/day vs. 22.8 g/day	0.61 (0.42, 0.87)
Smith et al., 2014 [51]; cohort (5 y)	Australia	General population; age 26–36	1386	FFQ	DSM-IV	≥2 times/week vs. <2 times/week	Men: 1.17 (0.74, 1.86) Women: 0.75 (0.57, 0.99)
Hamazaki et al., 2015 [26]; cross-sectional	Japan	University students; age 18–44	4190	FFQ	CES-D	Almost every day vs. almost never	0.65 (0.46, 0.92)
Mihrshahi et al., 2015 [48]; cohort (6 y)	Australia	Mid-age women; age 45–50	5117	FFQ	CES-D	>0 g/day vs. 0 g/day	0.89 (0.68, 1.17)
Wu et al., 2016 [36]; cross-sectional	Singapore	Senior ethnic Chinese residents of Singapore; age ≥55	2034	FFQ	GDS-15 (self-report)	≥3 times/week vs. ≤2 times/week	0.60 (0.40, 0.90)
Matsuoka et al., 2017 [47]; cohort (25 y)	Japan	General population; age 63–82	1181	FFQ	CES-D	152.6 g/day vs. 57.2 g/day	0.73 (0.41, 1.28)
Supartini et al., 2017 [34]; cross-sectional	Korea	General population; age 20–69	600	FFQ	CES-D	Frequently vs. occasionally	Men: 0.35 (0.11, 1.10) Women: 1.59 (0.52, 4.90)
Sánchez-Villegas et al., 2018 [30]; cross-sectional	Spain	General population; men age 55–75; women age 60–75	6587	FFQ	BDI-II, self-reported depression, use of antidepressants	155.28 g/day vs. 67.95 g/day	0.94 (0.77, 1.14)
Yang et al., 2018 [37]; cross-sectional	Korea	General population; age 19–64	9183	FFQ	Diagnosed with depression by a physician	≥4 times/week vs. <1 times/week	Men: 0.64 (0.30, 1.37) Women: 0.44 (0.29, 0.67)
Elstgeest et al., 2019 [41]; cohort (3, 6, 9 y)	Italy	General population; age 20–102	1058	FFQ	CES-D	Quartile4 vs. Quartile1	−0.97 (−1.74, −0.21)
Hamazaki et al., 2020 [43]; cohort (3 y)	Japan	Pregnant women	84,181	FFQ	EPDS	69.3 g/day vs. 5.2 g/day	0.84 (0.78, 0.90)
Sangsefidi et al., 2020 [31]; cross-sectional	Iran	General population; age 20–69	9965	FFQ	DASS 21	≥1 serving/week vs. never	1.54 (1.18, 2.01)
Ceolin et al., 2022 [24]; cross-sectional	Brazil	Elderly general population; age ≥60	1130	FFQ	GDS-15	twice a week or more vs. none	0.90 (0.81, 1.01)
Morales-Suárez-Varela et al., 2023 [28]; cross-sectional	Multicenter	University students	11,485	FFQ	Diagnosis of depression by a professional	Non-compliant vs. compliant	1.45 (1.28, 1.64)

Abbreviations: RR = Relative Risk; CI = Confidence Intervals; FFQ = Food Frequency Questionnaire; BDI = 21-item Beck Depression Inventory; BMI = body mass index; ATBC = Alpha-Tocopherol, Beta-Carotene Cancer Prevention; HSCL-25 = Hopkins Symptom Check List-25 subscale; CES-D = the Center for Epidemiological Studies Depression Scale; EPDS = the Edinburgh Postnatal Depression Scale; PRIME = the Prospective Epidemiological Study of Myocardial Infarction; MEDIS = the MEDiterranean Islands Elderly Study; GDS = Geriatric Depression Scale; CARDIA = Coronary Artery Risk Development in Young Adults; EPIC = the European Prospective Investigation Into Cancer and nutrition; SUN = Seguimiento Universidad de Navarra/University of Navarra Follow-up Project; RYUCHS = Ryukyus Child Health Study; M-CIDI = the Munich version of the Composite International Diagnostic Interview; CIDI-SF = the Composite International Diagnostic Interview Short-Form; ICD-10 = International Classification of Diseases (10th edition); CES-D-K = the Center for Epidemiological Studies Depression Scale, Korean version; SHLSET = Survey of Health and Living Status of the Elderly in Taiwan; KOMCHS = the Kyushu Okinawa Maternal and Child Health Study; CDAH = the Childhood Determinants of Adults Health; DSM-IV = Diagnostic and Statistical Manual of Mental Disorders, Fourth Edition; ALSWH = the Australian Longitudinal Study on Women’s Health; SLAS = the Singapore Longitudinal Aging Studies; JPHC = the Japan Public Health Center-based Prospective; PREDIMED = PREvencion con Dleta MEDiterranea; KNHANES = the Korea National Health and Nutrition Examination Survey; InCHIANTI = the Invecchiare in Chianti; JECS = the Japan Environment and Children’s Study; YaHS = Yazd Health Study; DASS = depression, anxiety.

**Table 2:** Summary of pooled risk estimates comparing fish consumption and depression risk.

	No. of Studies	RR (95% CI)	Heterogeneity		*p* for Difference
			*I*^2^ (%)	*p*	
Highest vs. lowest fish intake					
All studies	44	0.79 (0.73, 0.86)	66.5	<0.001	
Region					
Europe	18	0.75 (0.64, 0.87)	68.8	<0.001	0.084
Asia	14	0.75 (0.63, 0.90)	69.9	<0.001	
America	7	0.88 (0.80, 0.96)	0.0	0.473	
Multicenter	2	0.88 (0.53, 1.45)	92.9	<0.001	
Oceania	3	0.87 (0.71, 1.08)	26.4	0.257	
Pregnancy-relatedness					
Yes	5	0.78 (0.69, 0.89)	20.8	0.282	0.702
No	39	0.79 (0.72, 0.87)	69.1	<0.001	
Study design					
Cross-sectional	20	0.72 (0.63, 0.83)	79.0	<0.001	0.066
Case–control	1	0.54 (0.25, 1.19)	-	-	
Cohort	23	0.85 (0.78, 0.93)	31.5	0.076	
Gender					
Men and women	21	0.77 (0.68, 0.87)	76.9	<0.001	0.826
Men	9	0.81 (0.66, 0.99)	0.0	0.439	
Women	14	0.81 (0.71, 0.93)	61.1	0.001	
Only elderly population					
Yes	9	0.75 (0.62, 0.91)	79.5	<0.001	0.924
No	35	0.80 (0.73, 0.88)	61.9	<0.001	
Highest category of fish consumption ^a^					
Lower intake level	14	0.83 (0.69, 1.01)	80.0	<0.001	0.399
Higher intake level	14	0.75 (0.67, 0.84)	39.9	0.062	
Difference in fish intake between the highest and lowest categories ^b^					
Smaller intake difference	14	0.85 (0.72, 1.02)	77.9	<0.001	0.369
Larger intake difference	14	0.72 (0.63, 0.83)	53.2	0.010	
Increase of 15g/d (~1 serving/week)					
All studies	14	0.94 (0.92, 0.96)	36.0	0.088	
Region					
Europe	4	0.92 (0.88, 0.96)	0.0	0.929	0.216
Asia	7	0.94 (0.92, 0.97)	50.9	0.057	
America	3	0.89 (0.75, 1.04)	56.3	0.102	
Pregnancy-relatedness					
Yes	3	0.93 (0.89, 0.98)	59.6	0.084	0.079
No	11	0.94 (0.91, 0.96)	18.6	0.266	
Study design					
Cross-sectional	7	0.93 (0.90, 0.96)	29.4	0.204	0.072
Cohort	7	0.94 (0.92, 0.97)	30.1	0.198	
Gender					
Men and women	5	0.95 (0.93, 0.97)	0.0	0.931	0.588
Men	3	0.90 (0.80, 1.02)	39.7	0.190	
Women	6	0.91 (0.87, 0.96)	66.8	0.010	
Only elderly population					
Yes	2	0.96 (0.93, 0.99)	0.0	0.919	0.800
No	12	0.93 (0.90, 0.95)	45.7	0.042	

^a^ The value of 68.4 g corresponds to the median level of fish intake observed in the highest consumption category. ^b^ The value of 49.35 g represents the median difference in fish intake comparing the highest and lowest consumption categories.

## Data Availability

The original contributions presented in this study are included in the article/Supplementary Materials. Further inquiries can be directed to the corresponding author.

## Supplementary Materials

The following supporting information can be downloaded at: https://www.mdpi.com/article/10.3390/nu17243965/s1.

## Abbreviations

The abbreviations used throughout this manuscript are listed below:

WHO	World Health Organization
COVID-19	corona virus disease 19
PUFA	polyunsaturated fatty acid
RR	relative risk
HR	hazard ratio
OR	odds ratio
CI	confidence interval
FFQ	food frequency questionnaires
BMI	body mass index
