# Supplementary material for: Fish Consumption and the Risk of Depression: A Systematic Review and Meta-Analysis of Observational Studies
Source: Nutrients. 2025 Dec 18;17(24):3965. doi: 10.3390/nu17243965 (PMC12735933; doi:10.3390/nu17243965)
Supplement: Supplementary file 1 [file nutrients-17-03965-s001.zip › Supplementary Table S4.pdf]

Supplementary Table S4. Characteristics of observational studies included in the meta-analysis of fish consumption and the risk of depression

Author, year; study design (follow-up)	Country (study name)	Participants characteristics	No. of Subjects	Exposure assessment	Outcome measure	Amount of fish intake	RR (95% Cl) or β coefficient	Adjustment factors
Tanskanen et al., 2001; cross-sectional	Finland	General population; age: 25-64	3204	FFQ	BDI	Rare eaters vs. regular eaters	1.31 (1.10, 1.56)	Age, marital status, occupation, smoking status, physical activity, BMI, alcohol intake, coffee intake, educational level, and serum cholesterol level
Hakkarainen et al., 2004; cohort (5-8y)	Finland (ATBC study)	General population; age: 50-69	29133	FFQ	Hospital treatment	Quartile3 vs. Quartile1	0.97 (0.70, 1.33)	Age, BMI, energy intake, serum total and high-density lipoprotein cholesterol levels, consumption of alcohol, education, marriage, self-reported anxiety, self-reported depression, and smoking
Timonen et al., 2004; cohort (31y)	Finland (the Northern Finland 1966 birth cohort study)	General population; age: <31	5689	FFQ	HSCL-25 and diagnosis by medical doctor	regular eaters (weekly or more often) vs. rare eaters (monthly or more seldom)	Men: 0.8 (0.4, 1.6) Women: 2.4 (1.4, 4.2)	BMI, serum total cholesterol level, socioeconomic situation, alcohol intake, smoking, physical inactivity, and marital status
Barberger-Gateau et al., 2005; cross-sectional	France (the Three-City Study)	Community dwellers; age: ≥65	9280	FFQ	CES-D	>once a week vs. once a week	0.63 (0.52, 0.75)	Age, sex, education, and city
Miyake et al., 2006; cohort (2-9m)	Japan (the Osaka Maternal and Child Health Study)	Pregnant women; age: <32	865	FFQ	EPDS	72.9g/day vs. 23.1g/day	0.89 (0.50, 1.59)	Age, gestation, parity, cigarette smoking, family structure, family income, education, changes in diet in the previous month, season when data at baseline were collected, BMI, time of delivery before the second survey, medical problems in pregnancy,

								baby's sex and baby's birthweight
Appleton et al., 2007; cohort (5y)	Nothern Ireland and France (PRIME study)	General population (men); age: 50-59	10602	FFQ	Welsh Pure Depression subscale of the Minnesota Multiphasic Personality Inventory	Linear term	Nothern Ireland: -0.09 (-2.25, -0.01) France: -0.14 (-2.73, -1.17)	All diet and demographic variables
Astorg et al., 2008; cohort (2y)	France (SU.VI.MAX cohort study)	General population; age 35-60	3748	24-h dietary recall	Antidepressant or lithium prescription	Men: 87.9 ± 29.5g/day vs. 14.9 ± 8.9g/day Women: 71.6 ± 24.8g/day vs. 10.7 ± 7.1g/day	Men: 0.68 (0.38, 1.21) Women: 0.70 (0.48, 1.02)	Age, sex, intervention group (vitamin/mineral supplement or placebo), family status (living alone or in couple), education level, and tobacco use
Sontrop et al., 2008; cross-sectional	Canada (the Prenatal Health Project)	Pregnant women (10- and 22-week gestation)	2061	FFQ	CES-D	≥1serving/week vs. <1serving/week	-0.2 (-0.9, 0.4)	Sociodemographic, health and lifestyle variables
Bountziouka et al., 2009; cross-sectional	Greece and Cyprus (MEDIS study)	Elderly general population; age ≥65	1190	FFQ	GDS (self-report)	Linear term (1 portion of fish increase per week)	0.58 (0.45, 0.73)	Age, sex, education status, BMI, physical activity status, and the presence and management of hypertension, hypercholesterolemia, and diabetes
Colangelo et al., 2009; cohort (10y)	US (CARDIA study)	Generation population; age 24-42	3317	FFQ	CES-D	Quartile5 vs. Quartile1	Men: 0.89 (0.62, 1.28) Women: 0.75 (0.55, 1.01)	Age, race, gender, educational level, BMI, cigarettes per day, alcohol intake, total physical activity, marital status, employment status, income, and intakes of linoleic acid and folic acid, and energy intake
Kyrozis et al., 2009; cohort (6-13y)	Greece (the EPIC-Greece)	Elderly general population; age ≥60	610	FFQ	GDS	Linear term	-0.08 (-0.30, 0.15)	Age, gender, marital status, years of education, height, BMI, physical activity, smoking, alcohol intake, coffee intake, hypertension, diabetes

								mellitus, and energy intake
Sánchez-Villegas., 2009; cohort (4.4y)	Spain (SUN cohort)	General population; age 38 (mean)	10094	FFQ	Self-reported physician diagnosis of depression, anxiety or stress or use of antidepressant medication or tranquilizers	Quartile5 vs. Quartile1	0.85 (0 64, 1.13)	Age, sex, smoking status, BMI and its quadratic term, physical activity during leisure time, energy intake, and employment status
Strøm et al., 2009; cohort (1y)	Denmark (the Danish National Birth Cohort)	Women; age 25-40	54202	FFQ	Post-partum depression hospital admission or medicament prescription	0–3 g/day (1.1 g/day) vs. >30 g/day (38.0 g/day)	1.10 (0.87, 1.38)	Total energy intake, prepregnant BMI, maternal age, parity, alcohol intake, smoking, occupation, education, homeownership, marital status, social support, and history of previous depression
Murakami et al., 2010; cross-sectional	Japan (RYUCHS study)	Adolescents (school students); age 12-15	6517	FFQ	CES-D	29.1g/1000kcal vs. 9.1g/1000kcal	0.73 (0.55, 0.97)	Age, habitual exercise, paternal educational level, maternal educational level, living with father, living with mother, living with brother(s) or sister(s), number of siblings, municipality, BMI, and vegetable intake
Suominen-Taipale et al., 2010; cross-sectional	Finland (the Health 2000 Survey)	General population; age 45-74	5492	FFQ	M-CIDI	76g/day vs. 11g/day	0.6 (0.3, 0.9)	Age, total energy intake for fish consumption, BMI, level of education, marital status, smoking history, physical activity, alcohol intake for fish consumption, alcohol induced intoxication for consumption of fish, fish oil supplement use for fish consumption, occurrence of severe illness, bronchial asthma, or back pain or illness
Suominen-Taipale et al., 2010; cross-sectional	Finland (the Fishermen Study)	Fishermen with their families	1265	FFQ	CIDI-SF	Quartile4 vs. Quartile1	0.1 (0.02, 0.5)	Age, total energy intake for fish consumption, BMI, level of education, marital status, smoking history, physical activity, alcohol intake for fish consumption, alcohol induced

								intoxication for consumption of fish, fish oil supplement use for fish consumption, occurrence of severe illness, bronchial asthma, or back pain or illness
Chrysohoou et al., 2011; cross-sectional	Greece (IKARIA study)	Elderly population; age >65	673	FFQ	GDS (self-report)	≥3times week vs. never/rare	0.34 (0.19, 0.61)	Not specified
Li et al., 2011; cohort (10.6y)	US (the first National Health and Nutrition Examination Survey Follow-up Study)	General population; age 25-74	5068	FFQ	CES-D	Less than once a week vs. more than once a week	Men: 2.08 (1.08, 4.09) Women: 1.15 (0.83, 1.59)	Age, race/ethnicity, education attainment, family income level, marital status, types of residence area, occupation, and employment status, BMI, alcohol drinking, cigarette smoking, serum total cholesterol, total dietary energy intake, saturated fatty intake, frequency of eating fruit and vegetables, and self-evaluate health status and the history of major physical diseases (cancer, diabetes, stroke and heart attack)
Lucas et al., 2011; cohort (10y)	US (the Nurses' Health Study)	Nurses (women); age 50-77	54632	FFQ	Physician-diagnosed depression and regular antidepressant medication use	≥5 times/week vs. 1time/month	1.07 (0.74, 1.55)	Age, time interval of the study, hormonal status, race, obesity, smoking status, physical activity, diagnosis of diabetes, cancer, myocardial infarction, multivitamin use, average intake of energy, protein, trans fatty acids, saturated fatty acids, monounsaturated fatty acids, alcohol, n-3 and n-6-PUFA, and fish oil consumption
Albanese et al., 2012; cross-sectional	Multicenter (10/66 research program)	Community dwellers; age ≥65	14926	Standardized questions	ICD-10 depressive episode	Never eat fish vs. eat fish some days vs. eat fish most days	Never eat fish: 0.93 (0.78, 1.10) Eat fish some days: 1 (reference) Eat fish most days:	Age, gender, educational level, number of household assets, marital status, self-reported diagnosed diabetes, coronary heart disease and stroke, number of physical illnesses,

							1.07 (0.85, 1.36)	overall cognitive status, weekly meat intake, fruits and vegetables consumption, alcohol intake and physical activity level
Park et al., 2012; case-control	Korea	Patients diagnosed with a score ≥25 on the CES-D-K and controls without a chronic disease	80 patients and 88 controls	FFQ	CES-D-K	>9.62serving/ week vs. ≤2.57serving/ week	0.54 (0.19, 0.92)	Age, sex, drinking, marital status, sleeping hours, education, job, and energy except for energy intake
Tsai et al., 2012; cohort (5y)	Taiwan (SHLSET study)	Elderly population; age ≥65	1609	FFQ	CES-D	≥3times/week vs. <3times/week	0.91 (0.62, 1.14)	Age, gender, years of formal education, satisfaction with economic status, living setting, smoking status, alcohol drinking, betel-nut chewing, functional status, physical activity, cognitive status, and the presence of major chronic co-morbidities
Miyake et al., 2013; cross-sectional	Japan (KOMCHS)	Pregnant women	1745	FFQ	CES-D	71.7g/day vs. 22.8g/day	0.61 (0.42, 0.87)	Age, gestation, region of residence, number of children, family structure, history of depression, family history of depression, smoking, secondhand smoke exposure at home and at work, job type, household income, education, and BMI
Smith et al., 2014; cohort (5y)	Australia (CDAH study)	General population; age 26-36	1386	FFQ	DSM-IV	≥2times/week vs. <2times/week	Men: 1.17 (0.74, 1.86) Women: 0.75 (0.57, 0.99)	Men: Use of fish oil/ evening primrose oil supplements Women: Baseline marital status, smoking status, weight status, and self-reported health
Hamazaki et al., 2015; cross-sectional	Japan	University students; age 18-44	4190	FFQ	CES-D	Almost every day vs. almost never	0.65 (0.46, 0.92)	Age, sex, concerns over academic performance, friendships and financial matters, smoking status, consumption of alcohol and physical activity

Mihrshahi et al., 2015; cohort (6y)	Australia (ALSWH study)	Mid-age women; age 45-50	5117	FFQ	CES-D	>0g/day vs. 0g/day	0.89 (0.68, 1.17)	Education, marital status, BMI, physical activity, alcohol intake, fish and energy intake, smoking, and comorbidities
Wu et al., 2016; cross-sectional	Singapore (SLAS cohort)	Senior ethnic Chinese residents of Singapore; age≥55	2034	FFQ	GDS-15 (self-report)	at least three times per week vs. two or fewer times per week	0.60 (0.40, 0.90)	Age, sex, marital status, living, smoking, alcohol drinking, physical exercise, social and productive activities, self-rated health, hypertension, diabetes, heart failure or attack, stroke, fruits and vegetables, MMSE scores
Matsuoka et al., 2017; cohort (25y)	Japan (JPHC Study)	General population; age 63-82	1181	FFQ	CES-D	152.6g/day vs. 57.2g/day	0.73 (0.41, 1.28)	Age, sex, smoking status, alcohol frequency, physical activity, past history of depression, cancer, stroke, miocardial infarction, and diabetes mellitus
Supartini et al., 2017; cross-sectional	Korea	General population; age 20-69	600	FFQ	CES-D	Frequently vs. occasionally	Men: 0.35 (0.11, 1.10) Women: 1.59 (0.52, 4.90)	Age, sleep quality, exercise, smoked salmon, socio-demographic and health behavior variables
Sánchez-Villegas et al., 2018; cross-sectional	Spain (PREDIMED-PLUS trial)	General population; men age 55-75; women age 60-75	6587	FFQ	Self-reported life-time medical diagnosis depression or the habitual use of antidepressants, and BDI-II	155.28g/day vs. 67.95g/day	0.94 (0.77, 1.14)	Age, sex, marital status, educational level, smoking, and physical activity, BMI, hypercholesterolemia, hypertension, type 2 diabetes mellitus, energy intake, and adherence to the Mediterranean diet
Yang et al., 2018; cross-sectional	Korea (KNHANES)	General population; age 19-64	9183	FFQ	Diagnosed with depression by a physician	≥4times/week vs. <1times/week	Men: 0.64 (0.30, 1.37) Women: 0.44 (0.29, 0.67)	Age, sex, survey year, BMI, marital status, household income, education level, smoking status, alcohol consumption, physical activity, employment status, disease status, serum total cholesterol, energy intake, fruit consumption, vegetable consumption, red meat consumption, and white meat consumption

Elstgeest et al., 2019; cohort (3, 6, 9y)	Italy (InCHIANTI study)	General population; age 20-102	1058	FFQ	CES-D	Quartile4 vs. Quartile1	-0.97 (-1.74, -0.21)	Age, baseline CES-D score, sex, marital status, education level, physical activity, smoking, instrumental activities of daily living disabilities, alcohol intake and energy intake
Hamazaki et al., 2020; cohort (3y)	Japan (JECS)	Pregnant women	84181	FFQ	EPDS	69.3g/day vs. 5.2g/day	0.84 (0.78, 0.90)	Age, previous deliveries, BMI at 1 month after delivery, maternal highest educational level, annual household income, marital status at 6 months after delivery, alcohol intake at 1 month after delivery, smoking status at 1 month after delivery, physical activity during mid-late pregnancy, employment status during mid-late pregnancy for the analysis of postpartum depression at 6 months after delivery, employment status for the analysis of serious mental illness at 1 year after delivery, history of anxiety disorder, history of depression, sadness experienced during the past year, use of EPA and/or DHA supplementation, and presence of any congenital anomaly
Sangsefidi et al., 2020; cross-sectional	Iran (Yazd Health Study (YaHS))	General population; age 20-69	9965	FFQ	DASS 21	≥1serving/week vs. never	1.54 (1.18, 2.01)	Age, education level, physical activity level, history of chronic diseases, smoking, and BMI
Ceolin et al., 2022; cross-sectional	Brazil (EpiFloripa Aging cohort study)	Elderly general population; age≥60	1130	FFQ	GDS-15	twice a week or more vs. none	0.90 (0.81, 1.01)	Age, sex, education, leisure-time physical activity, BMI, dependence on activities of daily living and number of morbidities
Morales-Suárez-Varela et al., 2023; cross-	Multicenter (UniHcos project)	University students	11485	FFQ	Diagnosis of depression by a professional	Non-compliant vs. compliant	1.45 (1.28, 1.64)	Age, sex, BMI, housing, coexistence, marital status, and employment

sectional

Abbreviations: FFQ = Food Frequency Questionnaire; BDI = 21-item Beck Depression Inventory; BMI = body mass index; ATBC = Alpha-Tocopherol, Beta-Carotene Cancer Prevention; HSCL-25 = Hopkins Symptom Check List-25 subscale; CES-D = the Center for Epidemiological Studies Depression Scale; EPDS = the Edinburgh Postnatal Depression Scale; PRIME = the Prospective Epidemiological Study of Myocardial Infarction; MEDIS = the MEDiterranean Islands Elderly Study; GDS = Geriatric Depression Scale; CARDIA = Coronary Artery Risk Development in Young Adults; EPIC = the European Prospective Investigation Into Cancer and nutrition; SUN = Seguimiento Universidad de Navarra/University of Navarra Follow-up Project; RYUCHS = Ryukyus Child Health Study; M-CIDI = the Munich version of the Composite International Diagnostic Interview; CIDI-SF = the Composite International Diagnostic Interview Short-Form; ICD-10 = International Classification of Diseases (10th edition); CES-D-K = the Center for Epidemiological Studies Depression Scale, Korean version; SHLSET = Survey of Health and Living Status of the Elderly in Taiwan; KOMCHS = the Kyushu Okinawa Maternal and Child Health Study; CDAH = the Childhood Determinants of Adults Health; DSM-IV = Diagnostic and Statistical Manual of Mental Disorders, Fourth Edition; ALSWH = the Autralian Longitudinal Study on Women's Health; SLAS = the Singapore Longitudinal Aging Studies; JPHC = the Japan Public Health Center-based Prospective; PREDIMED = PREvencion con DIeta MEDiterranea; KNHANES = the Korea National Health and Nutrition Examination Survey; InCHIANTI = the Invecchiare in Chianti; JECS = the Japan Environment and Children's Study; YaHS = Yazd Health Study; DASS = depression, anxiety and stress scale questionnaire
