# Supplementary material for: Fish Consumption and the Risk of Depression: A Systematic Review and Meta-Analysis of Observational Studies
Source: Nutrients. 2025 Dec 18;17(24):3965. doi: 10.3390/nu17243965 (PMC12735933; doi:10.3390/nu17243965)
Supplement: Supplementary file 1 [file nutrients-17-03965-s001.zip › Supplementary Table S3.pdf]

Supplementary Table S3. Quality assessment of cohort studies

Study (Year)	Representativeness of the exposed cohort	Selection of the non exposed cohort	Ascertainment of exposure	Demonstration that outcome of interest was not present at start of study	Comparability of cohorts on the basis of the design or analysis §	Assessment of outcome	Was follow-up long enough for outcomes to occur †	Adequacy of follow up of cohorts ‡	No. of star
Hakkarainen et al., 2004	*	*	*	*	**	*	*	-	8
Timonen et al., 2004	*	*	*	*	*	*	*	*	9
Miyake et al., 2006	*	*	-	*	**	-	*	*	7
Appleton et al., 2007	*	*	*	*	*	-	*	-	6
Astorg et al., 2008	*	*	*	*	**	*	-	-	7
Colangelo et al., 2009	*	*	*	*	**	*	*	-	8
Kyrozis et al., 2009	*	*	*	*	**	*	*	*	9
Sánchez-Villegas., 2009	*	*	*	*	*	*	-	*	7
Strøm et al., 2009	*	*	*	*	**	*	*	-	8
Li et al., 2011	*	*	*	*	**	*	*	-	8
Lucas et al., 2011	-	*	*	*	*	*	*	-	6
Tsai et al., 2012	*	*	*	*	**	*	*	*	9
Smith et al., 2014	*	*	*	*	*	*	*	*	8
Mihrshahi et al., 2015	*	*	*	*	**	*	*	-	8
Matsuoka et al., 2017	*	*	*	*	*	*	*	-	7
Elstgeest et al., 2019	*	*	*	*	**	*	*	*	9
Hamazaki et al., 2020	*	*	*	*	**	*	*	*	9

A study could be awarded a maximum of one star for each item except for the item "Comparability". §A maximum of 2 stars could be awarded for this item. Studies that controlled for education level received one star, whereas studies that controlled for other important confounders such as body mass index, smoking status, or physical activity received an additional star. †A cohort study in which depression as the outcome with a follow-up time > 5 y was assigned one star, and a cohort study in which postpartum depression as the outcome with a follow-up time > 4 m was assigned one star. ‡A cohort study with a follow-up rate > 80% was assigned one star.
