# Supplementary material for: Fish Consumption and the Risk of Depression: A Systematic Review and Meta-Analysis of Observational Studies
Source: Nutrients. 2025 Dec 18;17(24):3965. doi: 10.3390/nu17243965 (PMC12735933; doi:10.3390/nu17243965)
Supplement: Supplementary file 1 [file nutrients-17-03965-s001.zip › Supplementary Table S2.pdf]

Supplementary Table S2. Quality assessment of case-control study

Study (Year)	Adequacy of the case definition	Representativeness of the cases	Selection of controls	Definition of controls	Comparability of cases and controls on the basis of the design or analysis §	Assessment of exposure	Same method of ascertainment for cases and controls	Non-response rate	No. of star
Park et al., 2012	*	*	*	*	**	*	*	-	8

A study could be awarded a maximum of one star for each item except for the item "Comparability". §A maximum of 2 stars could be awarded for this item. Studies that controlled for education level received one star, whereas studies that controlled for other important confounders such as body mass index, smoking status, or physical activity received an additional star.
