# Supplementary material for: Fish Consumption and the Risk of Depression: A Systematic Review and Meta-Analysis of Observational Studies
Source: Nutrients. 2025 Dec 18;17(24):3965. doi: 10.3390/nu17243965 (PMC12735933; doi:10.3390/nu17243965)
Supplement: Supplementary file 1 [file nutrients-17-03965-s001.zip › Supplementary Table S1.pdf]

Supplementary Table S1. Quality assessment of cross-sectional studies

Study (Year)	Representativeness of the sample	Sample size	Non-respondents	Ascertainment of the exposure (risk factor) §	Comparability §	Assessment of the outcome §	Statistical test	No. of star
Tanskanen et al., 2001	*	*	-	*	**	**	*	8
Barberger-Gateau et al., 2005	*	*	*	**	**	**	*	10
Sontrop et al., 2008	*	*	-	**	*	**	*	8
Bountziouka et al., 2009	*	*	*	*	**	*	*	8
Murakami et al., 2010	*	*	*	**	**	**	*	10
Suominen-Taipale et al., 2010 (health 2000)	*	*	*	**	**	**	*	10
Suominen-Taipale et al., 2010 (fishermen)	*	*	*	**	**	**	*	10
Chrysohoou et al., 2011	*	-	-	**	*	*	*	6
Albanese et al., 2012	*	*	-	**	**	**	*	9
Miyake et al., 2013	*	*	*	**	**	**	*	10
Hamazaki et al., 2015	*	*	*	**	*	**	*	9
Wu et al., 2016	*	*	*	*	*	*	*	7
Supartini et al., 2017	*	*	-	**	*	**	*	8
Sánchez-Villegas et al., 2018	*	*	*	**	**	**	*	10
Yang et al., 2018	*	*	*	**	**	**	*	10
Sangsefidi et al., 2020	*	*	*	**	**	**	*	10
Ceolin et al., 2022	*	*	*	**	**	**	*	10
Morales-Suárez-Varela et al., 2023	*	*	-	**	*	**	*	8

A study could be awarded a maximum of one star for each item except for the item "Ascertainment of the exposure", "Comparability", and "Assessment of outcome". §A maximum of 2 stars could be awarded for these items. Studies with no description of the measurement tool to assess the exposure received no star, whereas studies that used non-validated measurement tool, but the tool is available or described received one star, and studies that used validated measurement tool received two stars. Studies that controlled for education level received one star, and studies that controlled for any additional factor received an additional star. Studies with no description of outcome assessment method received no star, whereas studies that used self-reported outcome assessment method received one star, and studies that used independent blind assessment or record linkage method received two stars.
