# Supplementary figures and images for: Fish Consumption and the Risk of Depression: A Systematic Review and Meta-Analysis of Observational Studies
Source: Nutrients. 2025 Dec 18;17(24):3965. doi: 10.3390/nu17243965 (PMC12735933; doi:10.3390/nu17243965)

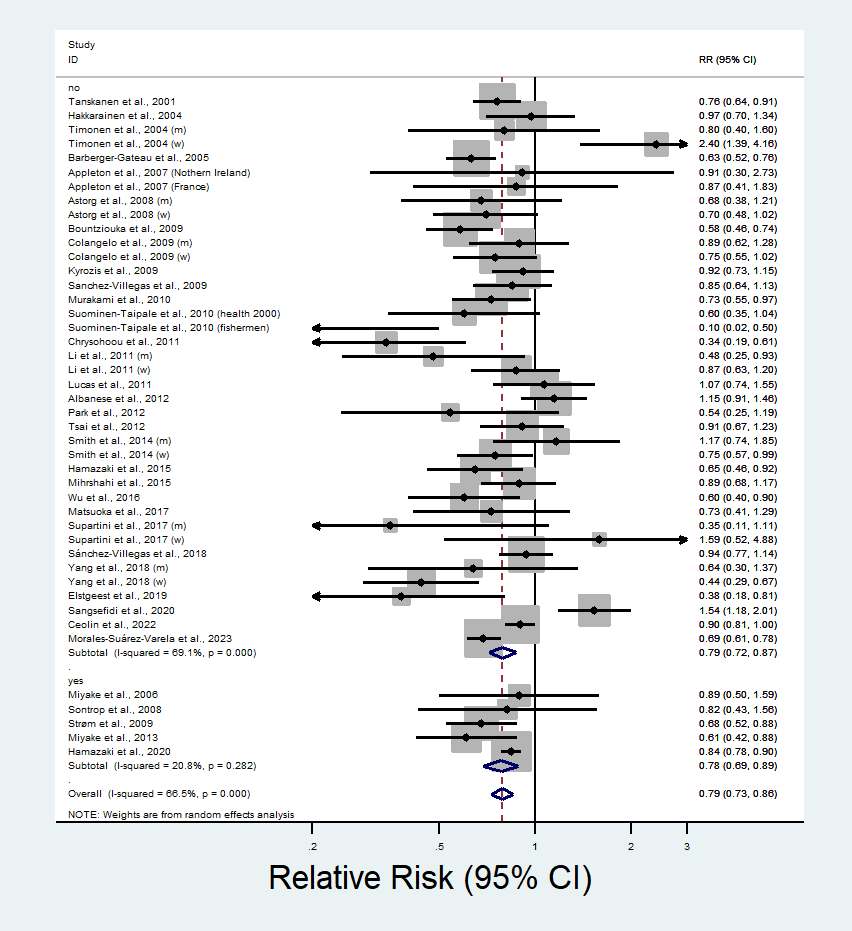

Supplement: Supplementary file 1 [file nutrients-17-03965-s001.zip › Supplementary Figure S1.png]
